# Effect of gestational weight gain on postpartum pelvic floor function in twin primiparas: a single-center retrospective study in China

**DOI:** 10.1186/s12884-023-05602-9

**Published:** 2023-04-20

**Authors:** Ying Zhou, Yetao Luo, Qirong Zhou, Jiangyang Xu, Shengyu Tian, Bizhen Liao

**Affiliations:** 1 Department of Obstetrics, The First Affiliated Hospital of Chongqing Medical University, No. 1, Youyilu Street, Yuzhong District, Chongqing, 400016 China; 2 Department of Nosocomial Infection Control, The Second Affiliated Hospital of Army Medical University, No. 83 Xinqiaozheng Street, Shapingba District, Chongqing, 400037 China

**Keywords:** Twin, Gestational weight gain, Pelvic floor disorders, Pelvic organ prolapse, Urinary incontinence

## Abstract

**Background:**

The effect of gestational weight gain (GWG) as a controllable factor during pregnancy pelvic floor function has rarely been investigated, and studies on twin primiparas are even less frequent. The objective of the present study was to explore the effect of GWG on postpartum pelvic floor function in twin primiparas.

**Methods:**

We retrospectively analyzed the clinical data of 184 twin primiparas in the pelvic floor rehabilitation system of the First Affiliated Hospital of Chongqing Medical University from January 2020 to October 2021. Based on the GWG criteria recommended by the Institute of Medicine, the study subjects were classified into two groups: adequate GWG and excessive GWG. Univariate and multivariate logistic regression models were applied to explore the relationship between GWG and pelvic floor function.

**Results:**

Among the 184 twin primiparas, 20 (10.87%) had excessive GWG. The rates of abnormal vaginal dynamic pressure (95% vs. 74.39%), injured type I muscle fibers (80% vs. 45.73%), anterior vaginal wall prolapse (90% vs. 68.90%), and stress urinary incontinence (50% vs. 20.12%) of twin primiparas with excessive GWG were significantly higher than those with adequate GWG. There was no significant difference between the total score of the Pelvic Floor Distress Inventory-Short Form 20 (PFDI-20) or the scores of the Pelvic Organ Prolapse Distress Inventory 6 (POPDI-6), the Colorectal-Anal Distress Inventory 8 (CRADI-8), and the Urinary Distress Inventory 6 (UDI-6) in the two groups (*P* > 0.05). After adjusting for potential confounding factors, the results showed that excessive GWG was positively associated with abnormal vaginal dynamic pressure (OR = 8.038, 95% CI: 1.001–64.514), injured type I muscle fibers (OR = 8.654, 95% CI: 2.462–30.416), anterior vaginal wall prolapse (OR = 4.705, 95% CI: 1.004–22.054), and stress urinary incontinence (OR = 4.424, 95% CI: 1.578–12.403).

**Conclusion:**

Excessive GWG in twin primiparas was positively correlated with the prevalence of pelvic floor dysfunction but did not exacerbate pelvic floor symptoms in twin primiparas. Controlling GWG within a reasonable range is recommended for reducing the risk of PFDs in pregnant women with twins.

## Background

In recent decades, the global rate of twin birth has been rising due to the delay of female childbearing age and the popularization of assisted reproductive technology [1]. Between 2012 and 2020, the birth rate of twins increased in China [2]. Compared with singletons, the incidence of postpartum pelvic floor injury and pelvic floor disorders (PFDs) is more significant in twin pregnant women [3, 4]. PFDs include pelvic organ prolapse (POP) and urinary incontinence (UI) [5], which are public health challenges for women worldwide and negatively affect the lives of millions of adult women [6–8]. The effect of weight factors on pelvic floor function has been a hot topic. Most studies have focused on the effects of maternal prepregnancy body mass index (BMI) and newborn birthweight on pelvic floor function [9–11], and few studies have explored the effects of GWG as a controllable factor during pregnancy on pelvic floor function. A systematic review and meta-analysis showed that excessive GWG is one of the risk factors for PFDs [12], and this limited study did not address the twin primiparas group.

With the increase in gestational age, GWG is a normal physiological process of pregnancy development [13]. Adequate GWG may lead to a reduction in the incidence of maternal and neonatal complications [14–16]. In 2009, the Institute of Medicine (IOM) published GMG guidelines and developed different criteria for GWG of singleton and twin pregnant women to promote controlling GWG to the normal range [17]. However, excessive GWG is becoming increasingly prevalent. A systematic review and meta-analysis that included more than one million pregnant women reported that approximately 51% of pregnant women gained more weight during pregnancy compared to the IOM recommendation [18]. Several studies from different regions of China have also found that more than 30% of Chinese women experienced more GWG in the third trimester than specified by the IOM criteria [19–22].

Excessive GWG in singleton women increases the risk of postpartum pelvic floor dysfunction. Compared to singleton pregnancies, twin pregnancies have more GWG due to the increased number of fetuses and physiological changes during pregnancy [17]. Based on this, we derived the following hypothesis: GWG of twin pregnancies excessively increases the risk of postpartum PFDs. Through retrospective analysis of the relationship between GWG and pelvic floor function in twin primiparas, this study aims to explore the effects of GWG on pelvic floor function in twin primiparas and provide a theoretical basis for reasonable control of pregnancy weight and prevention of PFDs.

## Method

### Study subjects

This study reviewed the postpartum pelvic floor function data of twin mothers in the postpartum pelvic floor rehabilitation system of the First Affiliated Hospital of Chongqing Medical University from January 2020 to October 2021. After matching with the electronic medical record system, according to the inclusion and exclusion criteria, 184 twin primiparas were finally included in the study. The inclusion criteria were twin primiparas with gestational weeks ≥ 34 weeks. Exclusion criteria: urinary incontinence before and during the pregnancy, pelvic organ prolapse before and during the pregnancy, multiparous women, singleton pregnancy, and multiple pregnancies other than twins. Since the IOM does not develop GWG criteria for twin pregnancies with prepregnancy underweight (BMI < 18.5), this group was excluded.

The 2009 IOM guidelines recommend that the normal range of GWG is 16.8–24.5 kg for twin pregnancies with normal prepregnancy BMI (BMI 18.5–24.9 kg/m^2^), 14.1–22.7 kg for those with prepregnancy overweight status (BMI 25–29.9 kg/m^2^), and 11.3–19.1 kg for those with prepregnancy obese status (BMI ≥ 30 kg/m^2^). These three groups were judged to have excessive GWG when their GWG exceeded 24.5, 22.7, and 19.1 kg, respectively. As there were no sample data with insufficient GWG in this study, the subjects were divided into two groups: adequate GWG and excessive GWG for analysis.

The study meets the ethical guidelines of the Helsinki Declaration and has been reviewed and approved by the Ethics Committee of the First Affiliated Hospital of Chongqing Medical University (2019–239). As a retrospective study, the Ethics Committee of the First Affiliated Hospital of Chongqing Medical University approved the exemption of informed consent, and the datasets were anonymized before their use.

### Data collection

The postpartum pelvic floor rehabilitation system recorded maternal data who of patients returned to the hospital for pelvic floor function examination after 6 weeks of delivery, including general maternal information, abdominopelvic pressure values measured by the instrument, POP results measured by the pelvic organ prolapse quantification (POP-Q) system, and the data filled in PFDI-20. The electronic medical record system includes demographic data and obstetric clinical data. The information in the two databases was matched by ‘hospitalization number (unique identity)’ using the VLOOKUP function in Excel 2019.

### Outcome assessment

The dynamic pressure value of the maximum vaginal contraction and pelvic floor muscle strength was measured by two fixed professionals using the PHENIX USB 4.0 (ELECTRONIC CONCEPT LIGNON INNOVATION, France) instrument. Before the measurement, the mother was instructed to empty her bladder and assume the lithotomy position. The examiner placed the zeroed electronic vaginal pressure balloon in the parturient's vagina, injected 5–10 ml gas into the balloon and fixed it, instructed the parturient to test synchronously as shown in the instrument, and read the value after the test.

A dynamic pressure value of 80–150 cm H_2_O for the maximum vaginal contraction was normal, and < 80 cm H_2_O was abnormal. Type I muscle fiber strength refers to the time that a muscle contraction can last with an intensity of more than 40% of its maximum value; it is graded by levels 0–V, where level 0 indicates contraction sustained for 0 s, level I indicates contraction sustained for 1 s, and so on, with level V meaning contraction sustained 5 s or more. Type II muscle fiber strength refers to the number of repeatable times when the maximum strength of muscle contraction reaches more than 60% and is signified by levels 0–V, where level 0 indicates achieved 0 times, level I indicates achieved 1 time, and so on, with level V indicating achieved 5 times or more. In accordance with previous literature [23, 24] and clinical practice, muscle strength levels 0-II were specified in analyses as defining injured pelvic floor muscle fibers.

POP is classified as anterior vaginal wall prolapse, apical vaginal prolapse (uterine prolapse), and posterior vaginal wall prolapse [25]. The objective diagnosis of POP is based on the evaluation results of the POP-Q examination, which is performed by a standardized trained pelvic floor rehabilitation therapist in accordance with the norms of a joint commission of the International Urogynecological Association (IUGA) and the International Continence Society (ICS) [26]. The hymen was taken as the fixed anatomical reference point in the POP-Q system, and its plane was defined as zero. There were six measurement points as follows: points Aa and Ba were the anterior vaginal wall sites, points C and D were the apical vaginal sites, and points Ap and Bp were the posterior vaginal wall sites. The anatomical position of the measuring point was negative above or near the hymen and positive below or distal. The examinee emptied the bladder, assumed a supine position, completed the Valsalva manoeuvre, and performed the examination when the maximum prolapse was reached. Aa point or Ba point values > -3 were diagnosed as indicative of anterior vaginal wall prolapse, C or D point values > 2—total vaginal length were diagnosed as indicative of apical vaginal prolapse or uterine prolapse, and Ap or Bp point values > -3 were diagnosed as indicative of posterior vaginal wall prolapse.

In this study, we adopted the validated Chinese version of the PFDI-20 questionnaire to investigate the effect of PFDs on the quality of life of postpartum women [27]. The PFDI-20 questionnaire comprises three subscales: POPDI-6, CRADI-8, and UDI-6. The total score of the three subscales was 0–300. The higher the score, the more severe the symptoms of PFDs and the greater the impact on maternal quality of life.

The existence of any type of UI is determined based on IUGA/ICS recommendations [25]. When the question ‘Do you usually experience urine leakage associated with a feeling of urgency, that is, a strong sensation of needing to go to the bathroom?’ was answered ‘Yes’, the diagnosis was urgent urinary incontinence (UUI). When the question ‘Do you usually experience urine leakage related to coughing, sneezing, or laughing?’ was answered ‘Yes’, the diagnosis was stress urinary incontinence (SUI). The diagnosis of mixed urinary incontinence (MUI) was made when ‘Yes’ was answered to both the aforementioned questions.

### Covariates

Through literature references, the covariates of this study included maternal demographic characteristics (age, prepregnancy BMI, education level, and place of residence), relevant surgical history (pelvic and abdominal surgery, in vitro fertilization, and embryo transfer [IVF-ET], cervical surgery), and obstetric clinical conditions (number of pregnancies, total birth weight of newborns, mode of delivery). Pelvic and abdominal surgery included appendectomy, adnexectomy, ovarian cystectomy, salpingotomy, myomectomy, and teratoma stripping. Cervical surgery included cervical conization and cervical polypectomy.

### Estimate sample size

We randomly collected 100 cases of twin primiparas as the preexperimental subjects, including 11 cases of excessive GWG. The incidence of SUI was 54.55% in the excessive GWG group and 20.22% in the adequate GWG group (*P* = 0.021). According to the pre-experimental results, the sample size was calculated by PASS15.0 software. At least 142 observations achieved 80% power at a 0.050 significance level to detect a change in probability (the prevalence rate of SUI) from the baseline value of 20.22% to 54.55%. This change corresponds to an odds ratio of 4.733.

### Statistical analyses

The data were analyzed and processed by SAS 9.4 software. Means ± standard deviations were used to describe normally distributed measures, and two independent samples t tests were used to compare the groups (adequate/excessive); median and interquartile range were used to describe measures of skewed distributions, and the Wilcoxon rank-sum test was used for comparison between the groups; the number of cases (n) and rates were used to describe the count data, and the χ^2^ test or Fisher's exact test was used to compare between the groups. The correlation between abnormal pelvic floor function and excessive GWG was explored using a multivariate logistic regression model. Power test of the multivariate logistic regression model in PASS 15.0. Two-sided *P* < 0.05 was considered statistically significant.

## Results

**Fig. 1:**
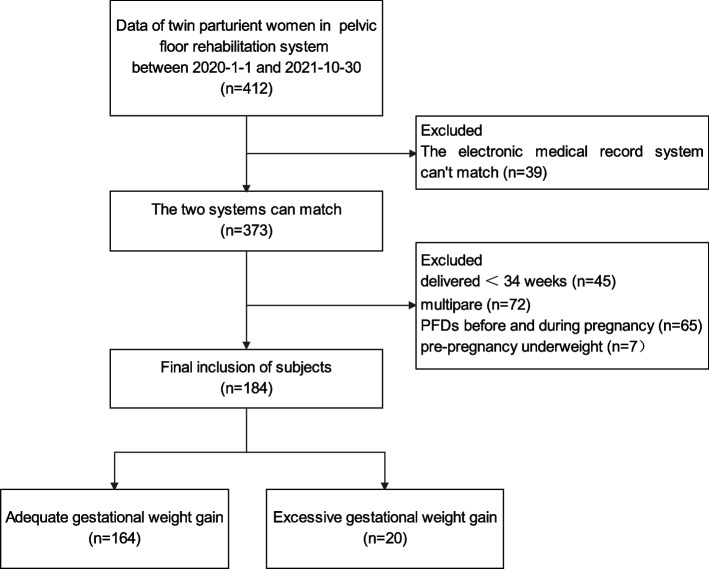
Flow chart of research subject selection

The maternal data from January 2020 to October 2021 of 412 twin pregnancies in the postpartum pelvic floor rehabilitation system were extracted. We excluded 39 cases whose electronic medical record system could not match, 72 cases of multiparas, 45 cases of delivery under 34 weeks, 65 cases of PFDs symptoms before and during pregnancy, and 7 cases of prepregnancy underweight. Finally, 184 cases of twin primiparas were included for analysis, including 164 in the adequate GWG group and 20 in the excessive GWG group (Fig. 1).

The basic characteristics and risk factors for the study population are shown in Table 1. The adequate GWG group included 164 (89.1%) subjects with a mean age of 30.3 ± 3.87 years; the excessive GWG group included 20 (10.9%) subjects with a mean age of 29.1 ± 4.44 years. There was no statistically significant difference between the two groups in terms of age, prepregnancy BMI, place of residence, history of pelvic and abdominal surgery, history of IVF-ET surgery, cervical surgery, number of pregnancies, gestational week at delivery, total birthweight of the newborns, or delivery mode (*P* > 0.05). BMI at delivery and GWG were higher in twin primiparas with excessive GMG than in twin primiparas with adequate GMG, and the difference was statistically significant (*P* < 0.05).

**Table 1:** Basic and clinical characteristics of the study population

Characteristic	Total (*n* = 184)	Adequate GWG (*n* = 164)	Excessive GWG (*n* = 20)	χ2/t/Z	*P* value
***Baseline demographic***					
**Age (years)**	30.17 ± 3.94	30.30 ± 3.87	29.10 ± 4.44	1.288	0.199
**Prepregnancy BMI (kg/m**^**2**^**)**	21.53 ± 2.55	21.61 ± 2.60	20.82 ± 2.00	1.322	0.188
**Education**					
Middle school and less	17 (9.24%)	15 (9.15%)	2 (10.00%)	0.043	0.979
High and secondary school	68 (36.96%)	61 (37.20%)	7 (35.00%)		
College and higher	99 (53.80%)	88 (53.66%)	11 (55.00%)		
**Residence**					
Rural	22 (11.96%)	20 (12.20%)	2 (10.00%)	—	> 0.999
Urban	162 (88.04%)	144 (87.80%)	18 (90.00%)		
***Relevant surgical history***					
**Pelvic or abdominal surgery**					
No	143 (77.72%)	127 (77.44%)	16 (80.00%)	—	> 0.999
Yes	41 (22.28%)	37 (22.56%)	4 (20.00%)		
**IVF-ET**					
No	93 (50.54%)	80 (48.78%)	13 (65.00%)	1.876	0.171
Yes	91 (49.46%)	84 (51.22%)	7 (35.00%)		
**Cervical surgery**					
No	179 (97.28%)	159 (96.95%)	20 (100.00%)	—	> 0.999
Yes	5 (2.72%)	5 (3.05%)	0 (0.00%)		
***Obstetrical clinical features***					
**Pregnancies**	2 (1,2)	2 (1,2)	1.5 (1,2)	0.173	0.863
**Gestation (weeks)**	37 (36.14,37.5)	37(36.14,37.43)	37.43(36.21,37.71)	1.664	0.096
**Total birthweight (g)**	5106.45 ± 599.58	5080.24 ± 563.14	5321.3 ± 829.03	-1.265	0.22
**BMI at delivery (kg/m**^**2**^**)**	28.41 ± 2.92	28.06 ± 2.76	31.28 ± 2.62	-4.947	** < 0.001**
**GWG (kg)**	17.6 ± 5.49	16.46 ± 4.55	26.95 ± 3.07	-13.568	** < 0.001**
**Delivery mode**					
Cesarean delivery	182 (98.91%)	162 (98.78%)	20 (100.00%)	—	> 0.999
Vaginal delivery	2 (1.09%)	2 (1.22%)	0 (0.00%)		

*Abbreviations*: *BMI* Body mass index, *GWG* Gestational weight gain, *IVF-ET* In vitro fertilization and embryo transfer

*P* < 0.05 are highlighted in bold text

Univariate analysis showed that the incidence values of SUI, abnormal vaginal dynamic pressure, injured type I muscle fibers, and anterior vaginal wall prolapse in the excessive GWG group were significantly higher than those in the adequate GWG group, *P* < 0.05 (Table 2).

**Table 2:** The relationship between GWG and pelvic floor function

Variable	Total (*n* = 184)	Adequate GWG (*n* = 164)	Excessive GWG (*n* = 20)	*P* value
**Abnormal vaginal dynamic pressure**	141 (76.63%)	122 (74.39%)	19 (95.00%)	**0.048**
**Injured muscle fibers**				
type I	91 (49.46%)	75 (45.73%)	16 (80.00%)	**0.004**
type II	116 (63.04%)	100 (60.98%)	16 (80.00%)	0.096
**Pelvic organ prolapse**				
Anterior vaginal wall prolapse	131 (71.20%)	113 (68.90%)	18 (90.00%)	**0.049**
Apical vaginal prolapse	34 (18.48%)	30 (18.29%)	4 (20.00%)	0.768
Posterior vaginal wall prolapse	30 (16.30%)	26 (15.85%)	4 (20.00%)	0.748
**Urinary incontinence**				
Stress urinary incontinence	43 (23.37%)	33 (20.12%)	10 (50.00%)	**0.009**
Urgent urinary incontinence	25 (13.59%)	22 (13.41%)	3 (15.00%)	0.739
Mixed urinary incontinence	11 (5.98%)	10 (6.10%)	1 (5.00%)	0.999

Abbreviations: *GWG* Gestational weight gain

*P* < 0.05 are highlighted in bold text

**Fig. 2:**
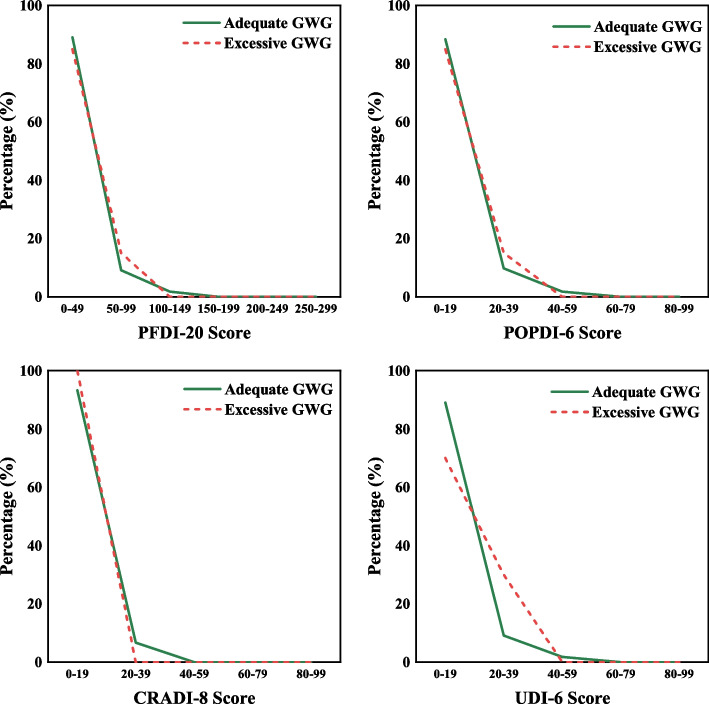
PFDI-20 and each subscale score distribution

Table 3 shows that the mean PFDI-20 score was 15.14 ± 23.90 in the group with adequate GWG and 14.01 ± 14.77 in the group with excessive GWG. There were no significant differences between the total score of the PFDI-20 and the scores of the subscales POPDI-6, CRADI-8, and UDI-6 in the two groups (*P* > 0.05). Figure 2 describes the distribution of the total PFDI-20 scores and the scores of each subscale for both adequate GWG and excessive GWG subjects. The distributions of the PFDI-20 and its subscale scores were very similar.

**Table 3:** The Correlation between GWG and PFDI-20

Variable	Total (*n* = 184)	Adequate GWG (*n* = 164)	Excessive GWG (*n* = 20)	t value	*P* value
POPDI-6	4.46 ± 8.69	4.47 ± 8.74	4.38 ± 8.49	0.047	0.963
CRADI-8	3.99 ± 8.11	4.12 ± 8.46	2.97 ± 4.24	0.994	0.326
UDI-6	6.57 ± 11.13	6.55 ± 11.24	6.67 ± 10.42	-0.042	0.966
PFDI-20	15.02 ± 23.06	15.14 ± 23.90	14.01 ± 14.77	0.298	0.767

*Abbreviations*: *PFDI-20* Pelvic Floor Distress Inventory—Short Form 20, *POPDI-6* Pelvic Organ Prolapse Distress Inventory 6, *CRADI-8* Colorectal Anal Distress Inventory 8, *UDI-6* Urinary Distress Inventory 6

A multivariate logistic regression model was used to explore the relationship between excessive GWG and pelvic floor dysfunction. Table 4 shows that after adjusting for age, prepregnancy BMI, education level, and place of residence, excessive GWG was significantly and positively correlated with abnormal vaginal dynamic pressure (OR = 8.128, 95% CI: 1.024–64.517), injured type I muscle fibers (OR = 7.153, 95% CI: 2.124–24.087), anterior vaginal wall prolapse (OR = 5.343, 95% CI: 1.152–24.778), and SUI (OR = 4.943, 95% CI: 1.800–13.577). Even after adding the relevant surgical history (pelvic and abdominal surgery, IVF-ET, cervical surgery) and obstetrical risk factors (number of pregnancies, total birthweights of newborns, delivery mode) into the regression model, abnormal dynamic vaginal pressure (OR = 8.038, 95% CI: 1.001–64.514), injured type I muscle fibers (OR = 8.654, 95% CI: 2.462–30.416), anterior vaginal wall prolapse (OR = 4.705, 95% CI: 1.004–22.054), and SUI (OR = 4.424, 95% CI: 1.578–12.403) were still associated with excessive GWG. The power of the logistic regression model of excessive GWG and abnormal vaginal dynamic pressure and anterior vaginal wall prolapse was 0.568–0.646. The power of the logistic regression model of excessive GWG and injured type I muscle fibers and SUI was 0.849–0.978 (Table 4).

**Table 4:** Results of multivariate logistic regression analysis of excessive GWG and abnormal pelvic floor function

Dependent variable	β	SE	Wald χ2	P value	OR (95% CI)	Power
**Model 1**^**a**^						
Abnormal vaginal dynamic pressure	2.095	1.057	3.930	**0.047**	8.128 (1.024, 64.517)	0.622
Injured type I muscle fibers	1.967	0.619	10.087	**0.001**	7.153 (2.124, 24.087)	0.970
Anterior vaginal wall prolapse	1.676	0.783	4.583	**0.032**	5.343 (1.152, 24.778)	0.646
Stress urinary incontinence	1.598	0.516	9.609	**0.002**	4.943 (1.800, 13.577)	0.905
**Model 2**^**b**^						
Abnormal vaginal dynamic pressure	2.097	1.058	3.928	**0.047**	8.144 (1.023, 64.814)	0.615
Injured type I muscle fibers	1.964	0.620	10.029	**0.002**	7.127 (2.114, 24.028)	0.968
Anterior vaginal wall prolapse	1.615	0.785	4.232	**0.040**	5.030 (1.079, 23.440)	0.612
Stress urinary incontinence	1.514	0.522	8.426	**0.004**	4.546 (1.635, 12.638)	0.868
**Model 3**^**c**^						
Abnormal vaginal dynamic pressure	2.084	1.063	3.847	**0.049**	8.038 (1.001, 64.514)	0.616
Injured type I muscle fibers	2.158	0.641	11.325	**0.001**	8.654 (2.462, 30.416)	0.978
Anterior vaginal wall prolapse	1.549	0.788	3.860	**0.049**	4.705 (1.004, 22.054)	0.568
Stress urinary incontinence	1.487	0.526	7.996	**0.005**	4.424 (1.578, 12.403)	0.849

*Abbreviations*: *GWG* Gestational weight gain

The independent variable is excessive GWG

*P* < 0.05 are highlighted in bold text

^a^Adjusted for age, prepregnancy BMI, education level, and place of residence

^b^Basis on Model 1, add pelvic and abdominal surgery, IVF-ET, and cervical surgery

^c^Basis on Model 2, add the number of pregnancies, the total weight of newborns, and delivery mode

## Discussion

The interaction among pelvic floor muscles, fascia, and ligaments is essential to support the normal physiological state and function of pelvic organs [28]. Damage to the pelvic floor muscle fibers can lead to structural defects and dysfunction of the pelvic floor, which in turn can lead to postpartum UI and POP [29–32]. We found that abnormal postpartum vaginal dynamic pressure and injuries to the pelvic floor type I and II muscle fibers were common phenomena in the twin pregnancy population regardless of whether GWG was adequate or excessive. Excessive GWG further increased the risk of pelvic floor muscle damage, with a high rate of abnormal vaginal dynamic pressure (95%, 19/20) and injured type I muscle fibers (80%, 16/20) in this group.

This study also found that, just as there was a higher proportion of abnormal dynamic vaginal pressure and injured type I muscle fibers in the excessive GWG group, there was also a higher incidence of postpartum anterior vaginal wall prolapse and SUI, the mechanism of which may be related to pelvic floor muscle damage due to excessive GWG [33]. During pregnancy, in the absence of pathological edema such as preeclampsia, heart failure, or nephropathy, excessive GWG is mainly associated with excessive maternal fat gain [13]. Excessive fat accumulation can create increasing and continuous pressure on the pelvic floor muscles and bladder, resulting in increased intra-abdominal pressure, leading to weakening of the pelvic floor muscle strength and destruction of fascia, causing damage to ligaments, nerves, blood vessels, and other tissues, thus changing the normal structure and anatomical position of the urethra and bladder and causing them to lose their original support, which represent the pathophysiological reasons for the occurrence of POP and SUI [34–36].

We found that the prevalence of postpartum anterior vaginal wall prolapse in twin primipara was 71.20% by the POP-Q examination, and the rate was as high as 90% in the group with excessive GWG. The occurrence of anterior vaginal wall prolapse was significantly associated with excessive GWG (OR = 4.705, 95% CI: 1.004–22.054). A randomized double-blind study [37] investigated 16,608 postmenopausal women with uteri, ages 50 to 79. Uterine prolapse, cystocele, and rectocele were evaluated using the Women's Health Plan prolapse classification system. Five-year follow-up data showed that the risk of prolapse progression in obese women as compared with the participants with healthy BMIs increased by 48% for cystocele, 58% for rectocele, and 69% for uterine prolapse, respectively. Young N et al. [38] performed an observational cross-sectional study. One thousand forty-three women were subjected to International Continence Society Pelvic Organ Prolapse Quantification prolapse assessment followed by 4D translabial ultrasonography. The study found that there is a positive association between BMI and posterior compartment prolapse on clinical examination and ultrasound imaging. Although the study methods were not entirely comparable, these findings may indicate that excessive weight gain can increase the risk of developing POP.

UI is a common disease that affects women's quality of life. SUI, UUI, and MUI are the main types, among which SUI is most common in postpartum women [39]. The results of this study showed that the incidence of postpartum SUI, UUI, and MUI in twin primiparas was 23.37%, 13.59%, and 5.98%, respectively, which was similar to the findings of a prospective longitudinal study conducted by Karen Ng et al. on singleton women [40]. We also found that SUI occurred in up to 50% (10/20) of twin primiparous women in the excessive GWG group, a rate which was significantly higher than that in the group with suitable GWG (P < 0.009). The prevalence of SUI was significantly positively correlated with GWG excess (OR = 4.424, 95% CI: 1.578–12.403). A systematic review and meta-analysis that included 46 studies with 73,010 subjects resulted in a similar conclusion: excessive GWG was associated with postpartum SUI [12]. We thus speculated that excessive GWG may play an etiological role in the development of SUI in twin primiparas.

Some studies have reported that the prevalence of postpartum SUI gradually decreases over time [41, 42]. Possibly due to differences in study populations and follow-up time, more studies have found the opposite: the effects of postpartum SUI on women's health are sustained, and over time, the cumulative incidence of SUI increases significantly [40, 43–47]. Other studies have found that the persistence of postpartum SUI is caused by higher BMI and excessive GWG in pregnant women [14, 40, 48, 49]. From these results, it is clear that control of excessive GWG is essential for the prevention of postpartum SUI. Preventive care for SUI should be provided throughout the life span of adult females in addition to during pregnancy and the puerperium period.

Kim BH et al. [50] calculated Pearson’s correlation coefficient for BMI and PFDI-20 and its subscales (POPDI-6, CRADI-8, and UDI-6). This study assessing the correlation between obesity and POP symptoms in Korean women found no statistically significant correlations between BMI and PFDI-20. This finding corroborates our results. Our study found that although excessive GWG was associated with an increased prevalence of PFDs, it did not further exacerbate pelvic floor symptoms in twin primiparas. This may be related to the small sample size of the excessive GWG group in this study or may be the reason for the effect of excessive GWG on pelvic floor symptoms no longer increasing after reaching a critical threshold level. The results of this study remind us that although excessive GWG does not exacerbate pelvic floor symptoms, pelvic floor injury and anatomical changes have occurred, and the negative impact of excessive GWG on the pelvic floor should not be overlooked as a result.

Although the birth rate of twins has increased significantly in recent decades, sample access in the case of twins is still limited compared to that of singletons. The above study sample was only from one tertiary teaching hospital in China, and the power of the logistic regression model of excessive GWG and injured type I muscle fibers and SUI was 0.849–0.978. This still needs to be verified by multicenter and large sample studies. In our study, urinary incontinence was assessed using only clinical symptoms, which may have the effect of reporter bias and judgments based on non-objective criteria. In a follow-up study, the diagnosis of urinary incontinence will be made by a combination of questionnaires and objective examinations. In assessing the effect of GWG on pelvic floor function, we only focused on the total growth of GWG and did not pay attention to the effect of the rate of weight gain at different periods of pregnancy. Additionally, this study did not explore postpartum pelvic floor function in the prepregnancy underweight and insufficient GWG groups, which will be the direction of follow-up research. The present study on the relationship between excessive GWG and pelvic floor function in twin primiparas is retrospective, and the argument for causality needs to be further confirmed by prospective cohort studies and randomized controlled trials.

## Conclusion

The results of this study showed that excessive GWG was positively associated with postpartum pelvic floor muscle impairment, anterior vaginal wall prolapse, and SUI in twin primiparous women, which is independent of other risk factors. Different from immutable factors such as age and race, GWG is a critical variable in prenatal care management because it is one of the few changeable factors affecting pelvic floor function. Prevention of excessive GWG is increasingly important for prenatal care providers and the functional health of the pregnant woman's pelvic floor.

## Data Availability

The datasets used and analyzed during the current study are available from the corresponding author on reasonable request.

## Abbreviations

GWG: Gestational weight gain
PFDI-20: Pelvic floor distress inventory-short form 20
POPDI-6: Pelvic organ prolapse distress inventory 6
CRADI-8: Colorectal anal distress inventory 8
UDI-6: Urinary distress inventory 6
PFDs: Pelvic floor disorders
POP: Pelvic organ prolapse
UI: Urinary incontinence
BMI: Body mass index
IOM: Institute of Medicine
POP–Q: Pelvic organ prolapse quantification
IUGA: International Urogynecological Association
ICS: The International Continence Society
UUI: Urgent urinary incontinence
SUI: Stress urinary incontinence
MUI: Mixed urinary incontinence
IVF-ET: In vitro fertilization and embryo transfer
